# Breast Cancer Screening in Saudi Arabia: Free but Almost No Takers

**DOI:** 10.1371/journal.pone.0119051

**Published:** 2015-03-16

**Authors:** Charbel El Bcheraoui, Mohammed Basulaiman, Shelley Wilson, Farah Daoud, Marwa Tuffaha, Mohammad A. AlMazroa, Ziad A. Memish, Mohammed Al Saeedi, Ali H. Mokdad

**Affiliations:** 1 Institute for Health Metrics and Evaluation, University of Washington, 2301 Fifth Ave., Suite 600, Seattle, Washington, United States of America; 2 Ministry of Health of the Kingdom of Saudi Arabia, Assadah, Al Murabba Riyadh, Saudi Arabia; ISPO, ITALY

## Abstract

**Introduction:**

Mammography ensures early diagnosis and a better chance for treatment and recovery from breast cancer. We conducted a national survey to investigate knowledge and practices of breast cancer screening among Saudi women aged 50 years or older in order to inform the breast cancer national health programs.

**Materials and Methods:**

The Saudi Health Interview Survey is a national multistage survey of individuals aged 15 years or older. The survey included questions on socio-demographic characteristics, tobacco consumption, diet, physical activity, health-care utilization, different health-related behaviors, and self-reported chronic conditions. Female respondents were asked about knowledge and practices of self and clinical breast exams, as well as mammography.

**Results:**

Between April and June 2013, a total of 10,735 participants completed the survey. Among respondents, 1,135 were women aged 50 years or older and were included in this analysis. About 89% of women reported not having a clinical breast exam in the past year, and 92% reported never having a mammogram. Women living in Al Sharqia had the highest rate of mammography use. Women who were educated, those who had received a routine medical exam within the last two years, and those who were diagnosed with hypertension were more likely to have had a mammogram in the past two years.

**Discussion:**

Our results show very low rates of breast cancer screening in the Kingdom of Saudi Arabia, a country with free health services. This calls for educational campaigns to improve breast cancer screening. Addressing the barriers for breast cancer screening is a public health imperative.

## Introduction

Breast cancer was the ninth leading cause of death for females in the Kingdom of Saudi Arabia (KSA) in 2010. [1,2] Moreover, 1,308 new breast cancer cases were reported in 2009, about 25% of all new cancer cases registered among Saudi women,[3] and it is expected that the incidence of breast cancer will increase over the coming decades in KSA due to the population’s growth and aging.[4]

Mammography ensures early diagnosis and a better chance for treatment and recovery.[5,6] The Centers for Disease Control and Prevention recommend mammography for breast cancer screening at least every two years for women aged 50–74 years old;[5] in KSA, this group formed 5.1% of the 20 million Saudis. Other types of screening are believed to be effective in detecting breast cancer, such as breast self-examination and clinical breast examination, but these are not recommended in updated guidelines for breast cancer screening.

In KSA, studies related to knowledge, attitudes, and practices around breast cancer are scarce. Milaat, in 2000, found a very low level of knowledge of breast cancer and its associated risk factors among female high-school students.[7] However, an older female population from Riyadh was found to be more knowledgeable about breast cancer. Among 864 women aged 20–50 years old and living in Riyadh, 82% knew about breast self-examination, and 61% knew about mammography. However, 41.2% had performed breast self-examination, and only 18.2% had ever had a mammogram.[8] In Al Hassa governorate, a population-based study found lower rates of mammography, 5.1% among 1,315 women aged 18–65 years old.[9] Another study of teachers in their thirties also showed low levels of breast-cancer-related knowledge, with only 32·4% being aware of breast self-examination.[10]

Whether Saudi women know about breast cancer screening and whether those above the age of 50 years are being screened for breast cancer at least once every two years is unknown. We conducted a national survey to investigate knowledge and practices of breast cancer screening among Saudi women aged 50 years or older in order to inform the breast cancer national health programs.

## Materials and Methods

### Ethics statement

The Saudi Ministry of Health and its IRB have approved the study protocol. The University of Washington IRB has deemed the study as IRB exempt since The Institute for Health Metrics and Evaluation received de-identified data for this analyses. All respondents consented and agreed to participate in the study. We used verbal consent that was captured by our computer program since it is commonly used and accepted in KSA. Two verbal consents were obtained: one for the household roster (obtained from the head of the household or the most knowledgeable person in the house) and another obtained from the randomly selected respondent. If the randomly selected respondent was between the ages of 15–17 years old, then the parent(s) or legal guardian of that individual consented on their behalf to participate in the study. The KSA Ministry of Health and The University of Washington IRB approved the verbal consents that were obtained in this study.

The Saudi Health Interview Survey is a national multistage survey of individuals aged 15 years or older. Households of Saudi citizens were randomly selected from a national sampling frame maintained and updated by the Census Bureau. The Ministry of Health divides KSA into 13 health regions, each with its own health department. We divided each region into sub-regions and blocks used by the KSA Department of Statistics. All regions were included, and a probability proportional to size was used to randomly select sub-regions and blocks. Households were randomly selected from each block. A roster of household members was collected, and an adult aged 15 or older was randomly selected to be surveyed.

The survey included questions on socio-demographic characteristics, tobacco consumption, diet, physical activity, health-care utilization, different health-related behaviors, and self-reported diagnosed chronic conditions. These conditions included hypertension, diabetes, and hypercholesterolemia.

Respondents were asked in which year they last visited a doctor or other health professional for a routine checkup. To assess self-rated health, they were asked, “In general, would you say your health is excellent, very good, good, fair, or poor?”

To assess knowledge of breast self-examination, women were asked, “Do you know what a self-breast examination is?” Those who answered yes were asked, “How many times during the past 12 months have you self-examined your breasts?” To assess type of professional breast cancer screening received, women were first asked, “How many times during the past 12 months have you visited a doctor’s office or other health professional for a (clinical) breast examination?” and then, “If you have ever had a mammography, in what year did you last have a mammography? A mammography is a screening test for breast tumors and cancers using a special type of x-ray.”

We examined knowledge and practices of breast self-exam over the last 12 months, clinical breast exam over the last 12 months, and time since last mammogram. We recognize that breast self-examination is not considered as an effective mean in the updated guidelines for breast cancer screening. However, since it is still being practiced in Saudi, we used it as a proxy to assess whether women are aware of the risk of breast cancer. We used a backward elimination multivariate logistic regression model to measure association between having received a mammogram for women aged 50 or older within the last two years with age, marital status, education, self-rated health, time since the last routine physical exam, and history of diagnoses with hypertension, diabetes, and hypercholesterolemia. Data were weighted to account for the probability of selection, and age and sex post-stratification based on census data for age and sex distribution of the Saudi population. We used probability proportional to size to select region and primary sampling units. Houses were randomly selected from each primary sampling unit. We applied a post stratification factor to account for non-response and to reflect the general KSA population. We created a final weight by using the inverse of the probability of selection multiplied by the post-stratification factor. Basically, we applied a standard methodology for creating weights similar to that of NHANES and BRFSS [11,12].We used SAS 9.3 for the analyses and to account for the complex sampling design.

## Results

Between April and June 2013, a total of 10,735 participants completed the SHIS out of 12,000 households originally contacted—a response rate of 89·4%. Among respondents, 1,001 were women aged 50–74 years old and were included in this analysis.

Only 25% of the female respondents aged 50–74 years old reported knowing about breast self-exam (Table 1). Among these, 57% reported performing a breast self-exam. About 89% of the women reported not having a clinical breast exam in the past year, and 92% of women aged 50–74 years old reported never having a mammogram.

**Table 1:** Knowledge and use of different breast cancer screening methods, Saudi women 50–74 years old, 2013.

Knowledge of what a self-breast exam is	Frequency	Weighted %	SE
No	741	74.9	2.0
Yes	185	25.1	2.0
**Self-breast exam over the last 12 months**			
0	72	42.8	5.0
1–3	65	34.3	4.5
4–6	11	6.9	2.4
7+	21	16.0	4.2
**Clinical breast exams received over the last 12 months**			
0	675	89.0	1.4
1	62	7.6	1.2
2–6	24	3.4	0.9
**Time since last mammogram**			
0–2 years ago	51	6.9	1.3
3–9 years ago	11	1.2	0.4
Never	820	91.9	1.3

SE = Standard Error.

Women who were educated, those who have received a routine medical exam within the last two years, and those who were diagnosed with hypertension were more likely to have had a mammogram in the past two years (Table 2).

**Table 2:** Use of mammography within the last two years by probable correlates, Saudi women 50–74 years old, 2013.

	Meeting guidelines for bi-yearly mammography screening						Backward elimination multivariate logistic regression	
	No			Yes				
Age	N	Weighted %	SE	N	Weighted %	SE	AOR	95% CI
50–59	499	93.7	1.4	31	6.3	1.4		
60–69	252	90.4	3.0	19	9.6	3.0	2.0	0.8–5.4
70+	83	99.0	1.0	1	1.0	1.0	0.2	0.0–2.0
**Marital status**								
Currently married	495	92.6	1.7	31	7.4	1.7		
Never married	15	100.0	0.0	0	.	0.0	NA	NA
Separated, divorced, or widowed	320	93.9	1.6	20	6.1	1.6	0.7	0.3–1.7
**Education**								
Primary school or less	732	94.1	1.4	33	5.9	1.4		
Elementary or high school completed	69	89.0	3.7	11	11.0	3.7	2.4	0.9–6.6
College degree or higher education	30	83.4	6.4	7	16.6	6.4	4.2	1.4–12.5
**Self-rated health**								
Excellent or very good	319	92.2	2.1	22	7.7	2.1		
Good	305	96.0	1.5	11	4.1	1.5	0.4	0.1–1.1
Fair or poor	209	90.6	3.2	17	9.4	3.2	1.2	0.4–3.6
**Time since last routine medical exam**								
Less than 2 years ago	277	89.3	2.2	31	10.2	2.2	2.5	1.2–5.2
2–3 years ago	15	100.0	0.0	0	0	.	NA	NA
4+ years ago	539	95.1	1.6	19	4.9	1.6		
**Diagnosis history of hypertension**								
No	565	94.9	1.2	29	5.1	1.2		
Yes	259	88.6	3.3	22	11.4	3.3	3.0	1.2–7.4
**Diagnosis history of diabetes**								
No	535	94.2	1.5	28	5.8	1.5		
Yes	289	90.7	2.3	23	9.3	2.3		
**Diagnosis history of hypercholesterolemia**								
No	630	93.8	1.5	28	6.2	1.5		
Yes	167	89.7	2.7	22	10.3	2.7		

SE = Standard Error, AOR = Adjusted Odds Ratio, 95% CI = 95% Confidence Interval; NA = Not Applicable.

**Fig 1:**
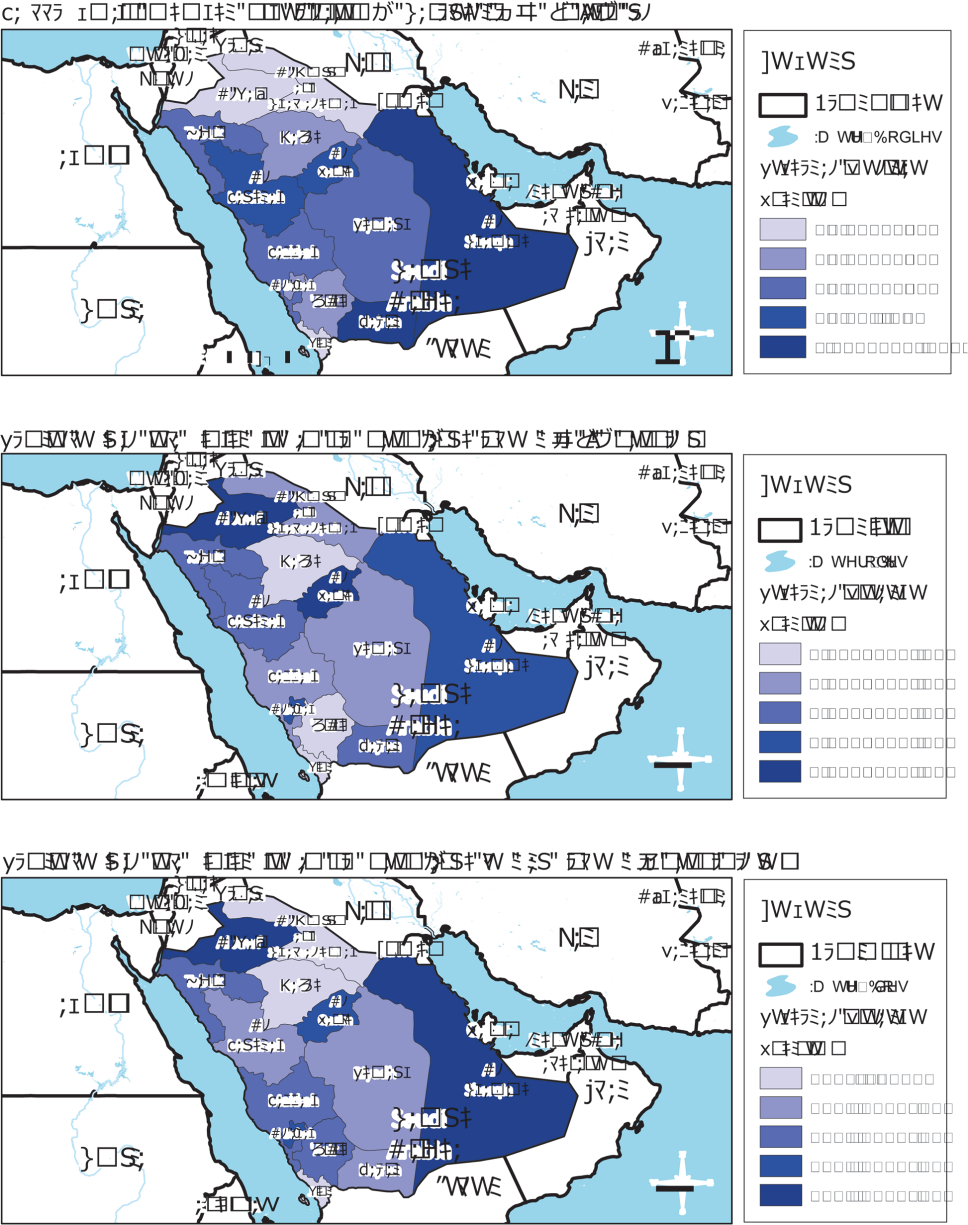
Regional rates of mammography during the last two years among Saudi women aged 50–74 years, and routine medical exam during the last two years among Saudi women aged 50–74 years and all Saudis aged 15 years or older.

Women who had a clinical breast exam and women who breastfed their last child for more than 12 months were more likely to have a mammogram (Table 3). Women living in the Al Sharqia region were the most likely to have had a mammogram within the last two years compared to women from all other Saudi regions (Fig. 1). Patterns of receiving a mammogram during the last two years mimicked those of reception of a routine medical exam among women aged 50–74 years old, and among the general population, by region (Fig. 1).

**Table 3:** Use of mammography within the last two years by clinical breast exam and duration of breastfeeding of last child, Saudi women 50–74 years old, 2013.

	Meeting guidelines for bi-yearly mammography screening					
	No			Yes		
	N	Weighted %	SE	N	Weighted %	SE
**Clinical breast exams received over the last 12 months**						
0	596	95.5	1.3	20	4.5	1.3
1	42	72.6	7.5	17	27.4	7.5
2–6	15	72.6	12.3	6	27.4	12.3
**Duration of breastfeeding the last child, in months**						
0	155	91.2	3.6	11	8.8	3.6
1–3	88	92.0	3.9	7	8.0	3.9
4–6	52	93.3	3.5	4	6.7	3.5
7–12	56	94.5	2.6	5	5.5	2.6
12+	94	86.0	5.5	9	14.0	5.5

SE = Standard Error.

## Discussion

Our study is the first to provide national estimates of breast cancer screening in the Kingdom of Saudi Arabia. Our results show a very low rate of breast screening in a country where health services are provided free of charge to the population. These findings are of great importance and call for immediate action to increase the rate of breast cancer screening in the Kingdom. It is crucial to identify barriers to seeking medical services and breast cancer screening in order to improve health outcomes.

Our data showed great geographic variations in breast cancer screening, ranging from 0.0–0.6% in Al Jawf and Al Hudud Ash Shamaliyah, to 9.7–17.0% in Najran and Al Sharqia. In our survey, 75% of respondents reported living less than 8 km from a health facility. Hence, access to a local health clinic for preventive care does not seem to be a factor. In addition, local health clinics have the ability to refer and transport patients in need of care to regional hospitals at no charge. All services provided at those clinics are free, and medications are dispensed free of charge.

KSA does not have a shortage of healthcare settings or providers. Due to increased oil revenues, a modern healthcare system was developed in KSA starting in 1970. In 1979, the country had 74 hospitals with 9,039 beds. By 2005, the number of hospitals reached 350, with nearly 48,000 beds, equaling about 1 bed per 500 residents, one of the lowest ratios in the world. These are operated by more than 34,000 physicians, of whom 80% are non-Saudis, and 70,000 nurses.[13] Also, healthcare is offered through more than 2000 local primary healthcare centers distributed over the country [14]. Mammography was introduced to KSA prior to 2002.[15] Specifically, a nationwide breast cancer screening center was established in Riyadh in 2007, and 1,215 were screened in the first year, although not all were 50 years or older at the time.[16] A regional mammography screening program, aimed at women 35–60 years old, was also established in 2007 in Al Qasim, and was preceded by an awareness campaign.[17] Mammography has been available in all regions of KSA since 2005. Our data showed that the highest rates of mammograms are in Al Sharqia, which indicates that the length of time mammography services have been available is not a driver of outcome. Indeed, if this were a factor, the highest rates would be in the capital city of Riyadh, where the earliest centers were established.

Despite their availability, mammography screening programs in KSA are opportunistic. Evidence shows that organized screening programs, ones with systematic call, recall, follow-up, and surveillance [18], not only utilize fewer resources, but are also more sensitive. Organized cancer screening programs have been shown to reduce cancer mortality, be more cost-effective, and have less harmful effects than opportunistic programs [19]. Therefore, transforming the current breast cancer screening programs in KSA into organized programs will reduce increase screening and reduce the burden of breast cancer.

Our findings should be viewed in the context of the KSA culture. Women are not allowed to drive and hence may be at a disadvantage to seek medical care. However, our findings rule out this possibility. Indeed, in some geographic areas women were more likely to seek routine medical exams than the general population.

Women in KSA are very conservative. They are more likely to shy away from preventive medical exams if these involve breast examination. However, all clinics in KSA have a female section that is operated by female nurses and physicians. Indeed, a study by Amin et al. found that traditions, mainly in the form of shyness and not wanting to be examined by a male physician, were the main reason for women not to seek a clinical breast exam.[9] It is possible that in certain facilities there are no female providers available to perform the exams and this may lead to lower screening levels. Shortage of specialized health facilities and shortage of female physicians was also cited by women in Amin et al’s study.[9] However, our data on regional patterns did not support this assumption of lack of female staff being a barrier to care, as mammography was higher in remote areas such as Najran compared to the capital of Riyadh, where one would expect more female physicians. Women’s perception of health services might be due to a lack of information on availability of services. It is possible that women think that female healthcare providers are lacking at health facilities, and hence, shy away from seeking a mammography. It is puzzling that women are not seeking such an effective and free medical service. Further qualitative studies are needed to understand the barriers to this life-saving exam, especially considering that most breast cancer patients in the Arab World have a more advanced stage of disease at first presentation, and their tumor size is larger, compared to European and American patients[20].

Our study has some limitations. First, all our variables are self-reported and may be subject to recall bias. Second, our sample for females 50 or older is very small to examine geographic variation in full detail. Indeed, the KSA population is young, with 78% under the age of 40,[21] and in our survey we did not oversample older age groups. However, our study is based on a national sample and covered all regions of KSA. Moreover, we conducted our surveys using standardized methods and techniques to ensure high-quality data.

Our results showed the same patterns of screening in KSA as we see elsewhere. In the United States, women who are more educated are more likely to have a mammogram.[22] However, in the United States, the rate of mammography during the last two years is 72·4% compared to 6·7% in KSA.[23] Indeed, KSA has a long way ahead until it reaches the levels of developed and economically advanced countries. However, we hope that the time it takes the Kingdom to reach such high levels is not prolonged.

It is possible that women in Saudi Arabia do not perceive breast cancer as a health danger. We have shown in a previous study that KSA has made tremendous improvement in health in the past 20 years[1,24]. It is very possible that health efforts in KSA have focused on infectious diseases and maternal and child health in the past years. Indeed, non-communicable diseases are now becoming the focus of the Ministry of Health, which recently developed and signed the Riyadh Declaration to increase the focus on non-communicable diseases.[25] Our findings call for accelerating these programs to improve breast cancer screening.

Empowering women is very crucial and a necessary part of improving their health. Unfortunately, most information on breast screening comes from screening campaigns. Although these campaigns and breast cancer awareness activities are currently widespread in KSA, knowledge about the disease is still very low among women. Moreover, information about breast cancer screening in the media is still scarce and not reaching all members of the community[26]. Indeed, the MOH and other health players in KSA have to consider more aggressive means to inform women about the importance of mammography. These means should include media, activists, and religious leaders among others. The advances in technology and messaging should be used to reach women everywhere. Moreover, perhaps it is time for a woman to champion this cause and be the spoke person to inform others to make the right health decisions. KSA is in dire need of female role models to improve the health of its female population. Indeed, it was shown that awareness campaigns based solely on advertising are not enough to produce mass screening and increase mammography in other Middle Eastern countries.[27] Women should be involved and given a voice to rally other around health and society issues to reduce the burden of disease. In the United States, most efforts to improve breast cancer screening were led by women activists who reached out to others and encouraged them to improve their health.[28] It is time for female leaders in KSA to champion breast cancer screening, among other causes.

Our study showed very low levels of breast cancer screening in KSA and calls for action to engage women and encourage them to seek and receive mammography. Furthermore, our data showed that screening rates are low in all segments of the population and in all regions of the Kingdom. This calls for national programs and educational campaigns. Explaining the benefits and the access to free mammography is crucial. Addressing the barriers such as lack of female health workers and educators is urgently needed. Indeed, programs designed, promoted, and implemented by regional female leaders are a public health imperative.

## References

[1] Mokdad AH, Jaber S, Aziz MIA, AlBuhairan F, AlGhaithi A, AlHamad NM, et al (2014) The state of health in the Arab world, 1990–2010: an analysis of the burden of diseases, injuries, and risk factors. Lancet 383: 309–320. 10.1016/S0140-6736(13)62189-3 24452042

[2] Lozano R, Naghavi M, Foreman K, Lim S, Shibuya K, Aboyans K, et al (2012) Global and regional mortality from 235 causes of death for 20 age groups in 1990 and 2010: a systematic analysis for the Global Burden of Disease Study 2010. The Lancet 380: 2095–2128. 10.1016/S0140-6736(12)61728-0 23245604 PMC10790329

[3] Saudi Cancer Registry (n.d.). Available: http://www.scr.org.sa/?module=publications&page=list&id=46&page_num=1. Accessed 2014 Nov 7.

[4] Ibrahim EM, Zeeneldin AA, Sadiq BB, Ezzat AA (2008) The present and the future of breast cancer burden in the Kingdom of Saudi Arabia. Med Oncol Northwood Lond Engl 25: 387–393. 10.1007/s12032-008-9051-5 18317955

[5] Tabár L, Gad A, Holmberg LH, Ljungquist U, Fagerberg CJG, Baldetorp L, et al (1985) Reduction in Mortality from Breast Cancer after Mass Screening with Mammography: Randomised Trial from the Breast Cancer Screening Working Group of the Swedish National Board of Health and Welfare. The Lancet 325: 829–832. 10.1016/S0140-6736(85)92204-4 2858707

[6] Verbeek ALM, Holland R, Sturmans F, Hendriks JHCL, Avunac M, Day NE, et al (1984) Reduction of Breast Cancer Mortality through Mass Screening with Modern Mammography: First Results of the Nijmegen Project, 1975–1981. The Lancet 323: 1222–1224. 10.1016/S0140-6736(84)91703-3 6144933

[7] Milaat WA (2000) Knowledge of secondary-school female students on breast cancer and breast self-examination in Jeddah, Saudi Arabia. East Mediterr Health J Rev Santé Méditerranée Orient Al-Majallah Al-Ṣiḥḥīyah Li-Sharq Al-Mutawassiṭ 6: 338–344. 11556021

[8] Aa A (2005) Knowledge of breast cancer and its risk and protective factors among women in Riyadh. Ann Saudi Med 26: 272–277. 10.5144/0256-4947.2006.272 PMC6074496 16883082

[9] Amin TT, Al Mulhim ARS, Al Meqihwi A (2009) Breast cancer knowledge, risk factors and screening among adult Saudi women in a primary health care setting. Asian Pac J Cancer Prev APJCP 10: 133–138. 19469641

[10] Dandash KF, Al-Mohaimeed A (2007) Knowledge, Attitudes, and Practices Surrounding Breast Cancer and Screening in Female Teachers of Buraidah, Saudi Arabia. Int J Health Sci 1: 61–71. 21475453 PMC3068667

[11] NHANES—National Health and Nutrition Examination Survey Homepage (n.d.). Available: http://www.cdc.gov/nchs/nhanes.htm. Accessed 2014 Nov 7.

[12] Centers for Disease Control and Prevention (CDC) (n.d.) Behavioral Risk Factor Surveillance System. Available: http://www.cdc.gov/brfss/. Accessed 2014 Nov 7.

[13] The Health Care Network, Royal Embassy of Saudi Arabia (n.d.). Available: http://www.saudiembassy.net/about/country-information/health_and_social_services/the_health_care_network.aspx. Accessed 2014 Nov 7.

[14] Saudi arabian monetary agency (SAMA) (n.d.) Kingdom of Saudi Arabia, Saudi arabian monetary agency forty ninth annual report: latest economic developments (2013). Available: http://www.sama.gov.sa/sites/samaen/ReportsStatistics/ReportsStatisticsLib/5600_R_Annual_En_49_Apx.pdf. Accessed 2014 Nov 7.

[15] Alkhenizan A, Hussain A, Alsayed A (2013) The sensitivity and specificity of screening mammography in primary care setting in Saudi Arabia. J Clin Oncol 31 Available: http://meetinglibrary.asco.org/content/117368-132. Accessed 8 August 2014.

[16] Abulkhair OA, Al Tahan FM, Young SE, Musaad SM, Jazieh A-RM (2010) The first national public breast cancer screening program in Saudi Arabia. Ann Saudi Med 30: 350–357. 10.4103/0256-4947.67078 20697170 PMC2941246

[17] Akhtar SS, Nadrah HM, Al-Habdan MA, El Gabbani SA, El Farouk GMK, Abdelgadir MH, et al (2010) First organized screening mammography programme in Saudi Arabia: preliminary analysis of pilot round. East Mediterr Health J Rev Santé Méditerranée Orient Al-Majallah Al-Ṣiḥḥīyah Li-Sharq Al-Mutawassiṭ 16: 1025–1031. 21222417

[18] Sankaranarayanan R, Budukh AM, Rajkumar R (2001) Effective screening programmes for cervical cancer in low- and middle-income developing countries. Bull World Health Organ 79: 954–962. 11693978 PMC2566667

[19] Miles A, Cockburn J, Smith RA, Wardle J (2004) A perspective from countries using organized screening programs. Cancer 101: 1201–1213. 10.1002/cncr.20505 15316915

[20] Chouchane L, Boussen H, Sastry KSR (2013) Breast cancer in Arab populations: molecular characteristics and disease management implications. Lancet Oncol 14: e417–e424. 10.1016/S1470-2045(13)70165-7 23993386

[21] Ramady MA (2010) The Saudi Arabian Economy: Policies, Achievements, and Challenges. 2nd ed. 2010 edition. New York: Springer. 534 p.

[22] Soni A (2007) Use of Breast Cancer Detection Exams among Women Age 40 and Over, U.S. Noninstitutionalized Population, 2005. Statistical Brief. Agency for Healthcare Research and Quality.

[23] Cancer Screening—United States, 2010 (n.d.). Available: http://www.cdc.gov/mmwr/preview/mmwrhtml/mm6103a1.htm. Accessed 2014 Aug 8.

[24] Memish ZA, Jaber S, Mokdad AH, AlMazroa MA, Murray CJL, Al Rabeeah AA, et al Burden of disease, injuries, and risk factors in the Kingdom of Saudi Arabia, 1990–2010. Prev Chronic Dis. 2014;11: E169 10.5888/pcd11.140176 25275806 PMC4184091

[25] International Conference on healthy Lifestyles and noncomunicable diseases in the arab world and the middle east. The Riyadh Declaration. (2012). Available: http://www.emro.who.int/images/stories/ncd/documents/Riyadh_Declaration.Final_bilingual.pdf?ua=1. Accessed 2014 Nov 7.

[26] Hagi SK, Khafiji MA (2013) Do Women in Saudi Arabia “Think Pink”? 1: American Journal of Research Communication.

[27] Adib SM, Sabbah MA, Hlais S, Hanna P (2009) Research in action: mammography utilization following breast cancer awareness campaigns in Lebanon 2002–05. East Mediterr Health J Rev Santé Méditerranée Orient Al-Majallah Al-Ṣiḥḥīyah Li-Sharq Al-Mutawassiṭ 15: 6–18. 19469422

[28] King S (2008) Pink Ribbons, Inc.: Breast Cancer and the Politics of Philanthropy. Minneapolis: Univ Of Minnesota Press 208 p.

