# Correction: Breast Cancer Screening in Saudi Arabia: Free but Almost No Takers

**DOI:** 10.1371/journal.pone.0124850

**Published:** 2015-04-08

**Authors:** The PLOS ONE Staff

There are a number of errors in Fig 1. The authors have provided a corrected version here. The publisher apologizes for the errors that were introduced during the typesetting process.

**Fig 1:**
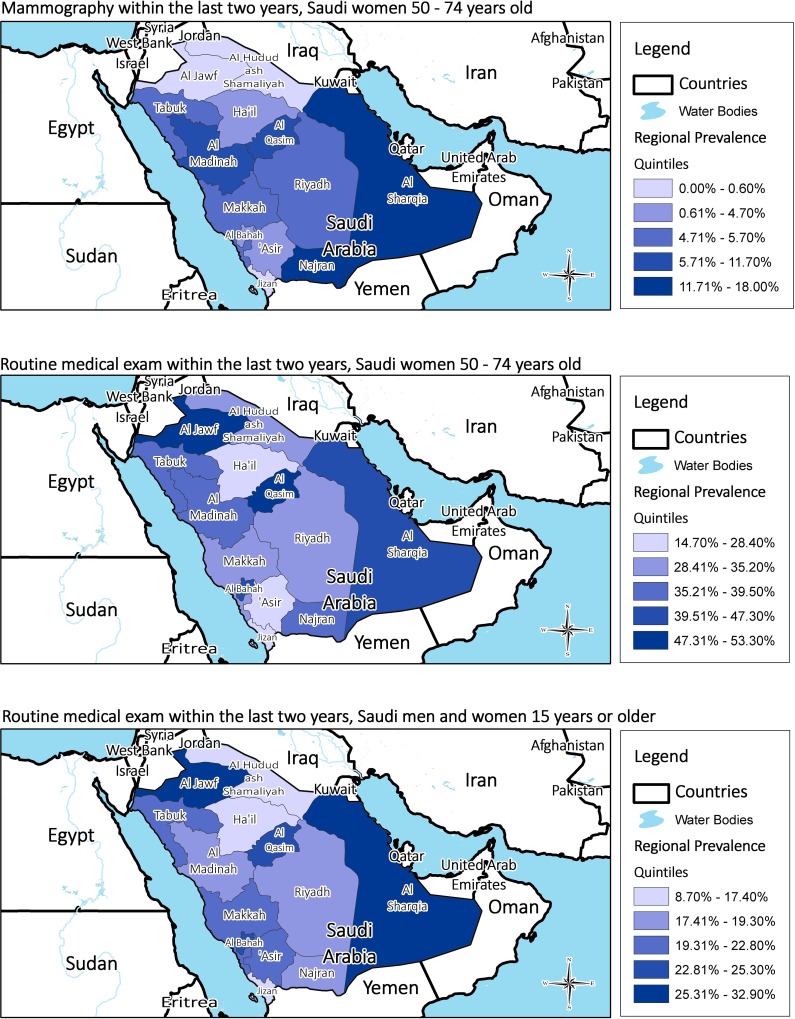
Regional rates of mammography during the last two years among Saudi women aged 50–74 years, and routine medical exam during the last two years among Saudi women aged 50–74 years and all Saudis aged 15 years or older.
